# Acute Inflammation Confers Enhanced Protection against Mycobacterium tuberculosis Infection in Mice

**DOI:** 10.1128/spectrum.00016-21

**Published:** 2021-07-07

**Authors:** Tucker J. Piergallini, Julia M. Scordo, Paula A. Pino, Larry S. Schlesinger, Jordi B. Torrelles, Joanne Turner

**Affiliations:** a Host-Pathogen Interactions Program, Texas Biomedical Research Institute, San Antonio, Texas, USA; b Biomedical Sciences Graduate Program, The Ohio State University, Columbus, Ohio, USA; c The Barshop Institute, The University of Texas Health Science Center at San Antonio, San Antonio, Texas, USA; d Population Health Program, Texas Biomedical Research Institute, San Antonio, Texas, USA; Emory University School of Medicine

**Keywords:** *Mycobacterium tuberculosis*, inflammation, neutrophils, lipopolysaccharide, LPS

## Abstract

Inflammation plays a crucial role in the control of Mycobacterium tuberculosis infection. In this study, we demonstrate that an inflammatory pulmonary environment at the time of infection mediated by lipopolysaccharide treatment in mice confers enhanced protection against M. tuberculosis for up to 6 months postinfection. This early and transient inflammatory environment was associated with a neutrophil and CD11b^+^ cell influx and increased inflammatory cytokines. *In vitro* infection demonstrated that neutrophils from lipopolysaccharide-treated mice exhibited increased association with M. tuberculosis and had a greater innate capacity for killing M. tuberculosis. Finally, partial depletion of neutrophils in lipopolysaccharide-treated mice showed an increase in M. tuberculosis burden, suggesting neutrophils played a part in the protection observed in lipopolysaccharide-treated mice. These results indicate a positive role for an inflammatory environment in the initial stages of M. tuberculosis infection and suggest that acute inflammation at the time of M. tuberculosis infection can positively alter disease outcome.

**IMPORTANCE** Mycobacterium tuberculosis, the causative agent of tuberculosis disease, is estimated to infect one-fourth of the world’s population and is one of the leading causes of death due to an infectious disease worldwide. The high-level variability in tuberculosis disease responses in the human populace may be linked to immune processes related to inflammation. In many cases, inflammation appears to exasperate tuberculosis responses; however, some evidence suggests inflammatory processes improve control of M. tuberculosis infection. Here, we show an acute inflammatory stimulus in mice provides protection against M. tuberculosis for up to 6 months, suggesting acute inflammation can positively affect M. tuberculosis infection outcome.

## INTRODUCTION

Tuberculosis (TB) disease, caused by the bacterium Mycobacterium tuberculosis, is a global health burden. In 2019, 1.4 million people died from TB, making TB the leading cause of death due to an infectious disease worldwide in that year (1). There is variability in M. tuberculosis infection outcomes among individuals, with some maintaining infection in a latent state (M. tuberculosis latency) and others developing active TB disease (1). Although individual responses to TB in humans and animal models have been characterized in many studies, the exact reasons why one host will respond differently from another is unknown.

Worse TB outcomes are associated with significant amounts of overall inflammation and host-directed damage (2), indicating that inflammation on its own may exacerbate M. tuberculosis infection. However, numerous studies have indicated the need for basic inflammatory processes in the control of TB disease, and others have shown enhanced inflammation can increase M. tuberculosis protection in various *in vitro* and *in vivo* models (3–11). Therefore, the role of inflammation in TB disease is not resolved. Comorbidities associated with increased TB susceptibility in humans are also associated with increased inflammation. HIV infection, while primarily conferring susceptibility to TB via depletion of CD4 T cells, is also associated with an increase in the overall inflammatory state (12–14). Diabetes increases active TB risk and itself is associated with systemically increased inflammation (15, 16). Chronic obstructive pulmonary disease (COPD) and smoking also are risk factors for TB and are also associated with inflammation (17–19). Another TB comorbidity, natural aging, can predispose individuals to reactivation of latent M. tuberculosis infection as well as increase TB disease mortality (20) and is associated with an increased pulmonary and systemic inflammatory state known as inflammaging (21–23). However, aged mice show an early control of M. tuberculosis infection associated with cellular inflammation, and while this is only transient, it does suggest that enhanced inflammation early helps control M. tuberculosis infection (8–11, 24). Other studies have also indicated enhanced inflammation and cytokine secretion can improve mycobacterial control (3–11). Additionally, the observation that some individuals may be capable of early clearance of TB suggests that a robust early inflammatory response can mediate control of M. tuberculosis or at least an alteration in how latent M. tuberculosis infection is established (25, 26). How inflammation can protect or worsen TB disease in these highly variably disease states and models is unknown and warrants further study to better understand the variable outcomes of TB.

To determine how acute inflammation can influence M. tuberculosis infection outcome, we utilized short-term low-dose lipopolysaccharide (LPS) treatment in mice to generate an increased acute systemic and pulmonary inflammatory response at the time of M. tuberculosis infection. LPS treatment caused an increase of inflammatory cytokines and myeloid cells, primarily neutrophils and CD11b^+^ cells, in the mouse lungs. Following M. tuberculosis infection, LPS-treated mice had a significant reduction of M. tuberculosis burden evident as early as 7 days postinfection, an effect that persisted for at least 6 months. *In vitro* analyses suggested neutrophils play a role in early M. tuberculosis control through *in vivo* depletion of neutrophils in LPS-treated mice prior to M. tuberculosis infection, although the contribution of other immune cells in the lung cannot be ruled out. Our findings confirm that a transient, acute, increased inflammatory environment at the time of M. tuberculosis entry into the lung can impact the course of infection by reducing M. tuberculosis burden in the lung, adding further information to discern why M. tuberculosis-exposed people have different infection and disease outcomes.

## RESULTS

### LPS causes pulmonary and systemically increased inflammation in mice.

**FIG 1:**
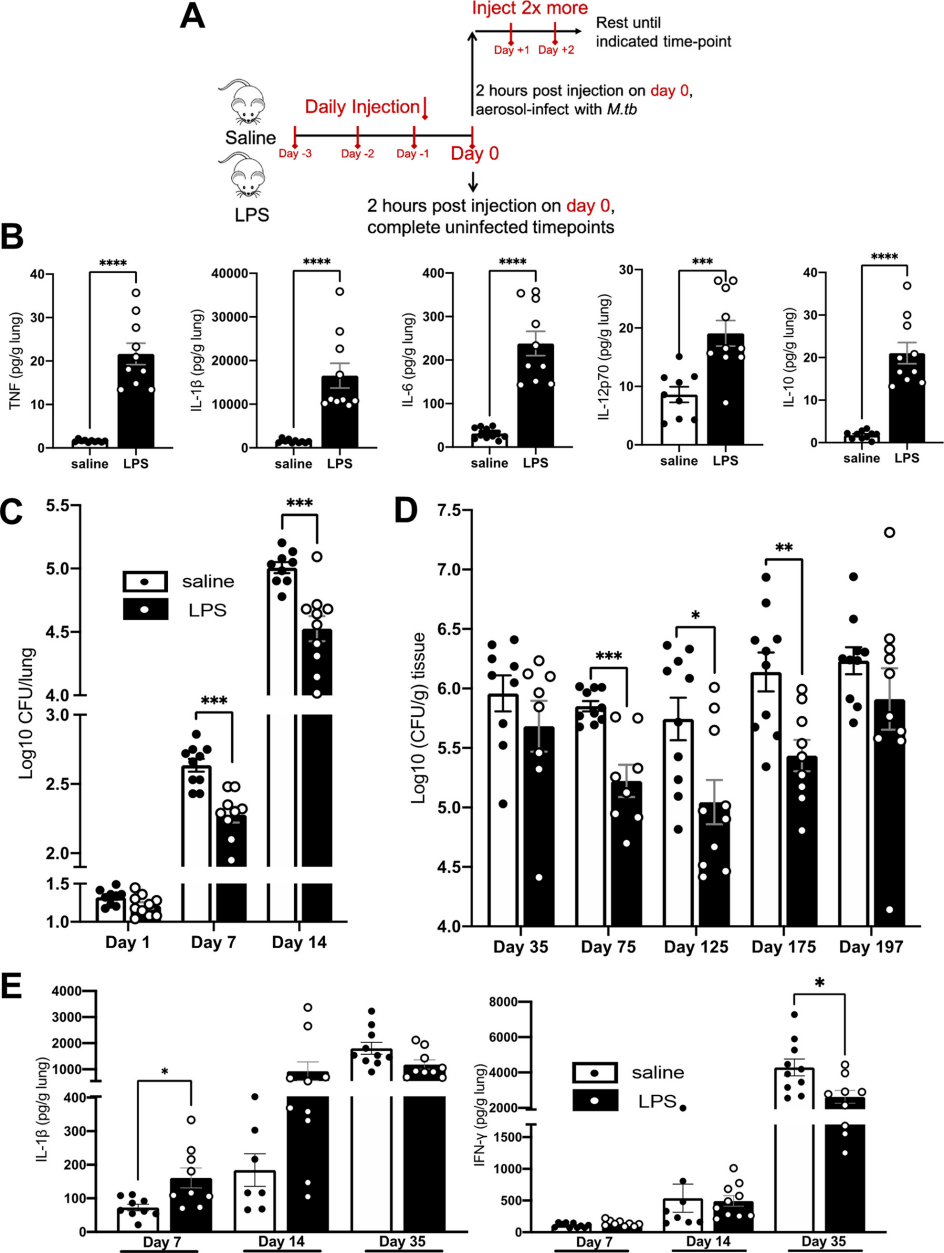
Cytokines and CFU in LPS mice. (A) Schematic of injection scheme. (B) Male BALB/c mice were injected with LPS as described in the text. On day 0, lungs were isolated and protein content determined via Luminex, normalized to organ mass. Protein levels of TNF, IL-1β, IL-6, IL-12p70, and IL-10 are shown. (C and D) LPS or saline BALB/c mice were aerosol infected with M. tuberculosis as described in the text. CFU burdens at the indicated time point of whole (C) or partial (D) lungs from male (C) and female (D) mice are shown. Partial lung CFU numbers were normalized to lung mass. (E) At the indicated time point, protein levels via ELISA of IL-1β and IFN-γ in male BALB/c mice are shown. Data are representative of 2 independent experiments of 4 or 5 mice in each group. Unpaired Student's *t* test, *, *P* < 0.05; **, *P* < 0.01; ***, *P* < 0.001; ****, *P* < 0.0001.

To evaluate the impact of acute inflammation on M. tuberculosis infection, we injected male BALB/c mice with LPS or saline via the intraperitoneal (i.p.) route every 24 h, for 4 total injections (Fig. 1A). Two hours after the fourth injection, termed day 0, we analyzed the local inflammatory response in the lungs. Tumor necrosis factor (TNF), interleukin-1beta (IL-1β), IL-6, IL-12p70, and IL-10 were significantly increased in the lungs of LPS-injected mice (LPS mice) (Fig. 1B). We also saw a concomitant increase of TNF, IL-1β, IL-6, and IL-10 in the spleens of LPS mice, while IL-12p70 showed no differences (see Fig. S1A in the supplemental material). C-reactive protein (CRP) levels showed no difference in the lung (Fig. S1B) but was increased in spleen and liver (Fig. S1B). To confirm LPS affected different mouse sexes and strains the same, we injected female BALB/c and male C57BL/6 mice with LPS and saw the same increase in lung inflammation as that observed in male BALB/c mice, with the exception that IL-12p70 was not increased in male C57BL/6 lungs (Fig. S1C and D).

### Acute inflammation protects mice from M. tuberculosis infection.

We infected male BALB/c LPS mice with M. tuberculosis on day 0 (4 days after initiation of LPS/saline treatment), injected with LPS/saline daily for 2 more days, and rested until the indicated time points (Fig. 1A). At 1 day postinfection (dpi), we found no difference in CFU numbers (Fig. 1C), whereas at 7 and 14 dpi we saw significantly fewer CFU (∼0.5 log_10_) in LPS mice (Fig. 1C). To determine if the early control of M. tuberculosis was sex or mouse strain specific, we repeated these studies in female BALB/c mice and male C57BL/6 mice, showing that at 14 dpi, female BALB/c and male C57BL/6 LPS mice also had significantly fewer CFU than saline-injected mice (saline mice) (Fig. S2A and B). These results suggest that the effects of LPS on the early control of M. tuberculosis was not sex or strain specific but was related to LPS-induced inflammation. We further determined the CFU content at 35, 75, 125, 175, and 197 dpi and observed lower CFU levels (∼0.25, ∼0.6, ∼1.0, ∼1.0, ∼0.4 log_10_ [CFU/g tissue], respectively) in LPS mice at all time points (Fig. 1D), although only days 75, 125, and 175 showed statistical significance.

When determining cytokine protein levels at 7, 14, and 35 dpi, we observed that IL-1β levels in the lung were elevated at 7 dpi, with a trend increase at 14 dpi (Fig. 1E). Gamma interferon (IFN-γ) levels in lung showed no differences at 7 and 14 dpi (Fig. 1E). TNF levels showed no differences at 7 dpi but showed a trend increase at 14 dpi compared to saline (Fig. S2C). At 35 dpi, LPS mouse lungs showed significantly less IFN-γ and a decrease of IL-1β and TNF, likely a result of their lower level of M. tuberculosis CFU burden at this time compared to saline-treated M. tuberculosis-infected mice (Fig. 1E and Fig. S2C).

### LPS mice have more activated myeloid cells in the lungs.

**FIG 2:**
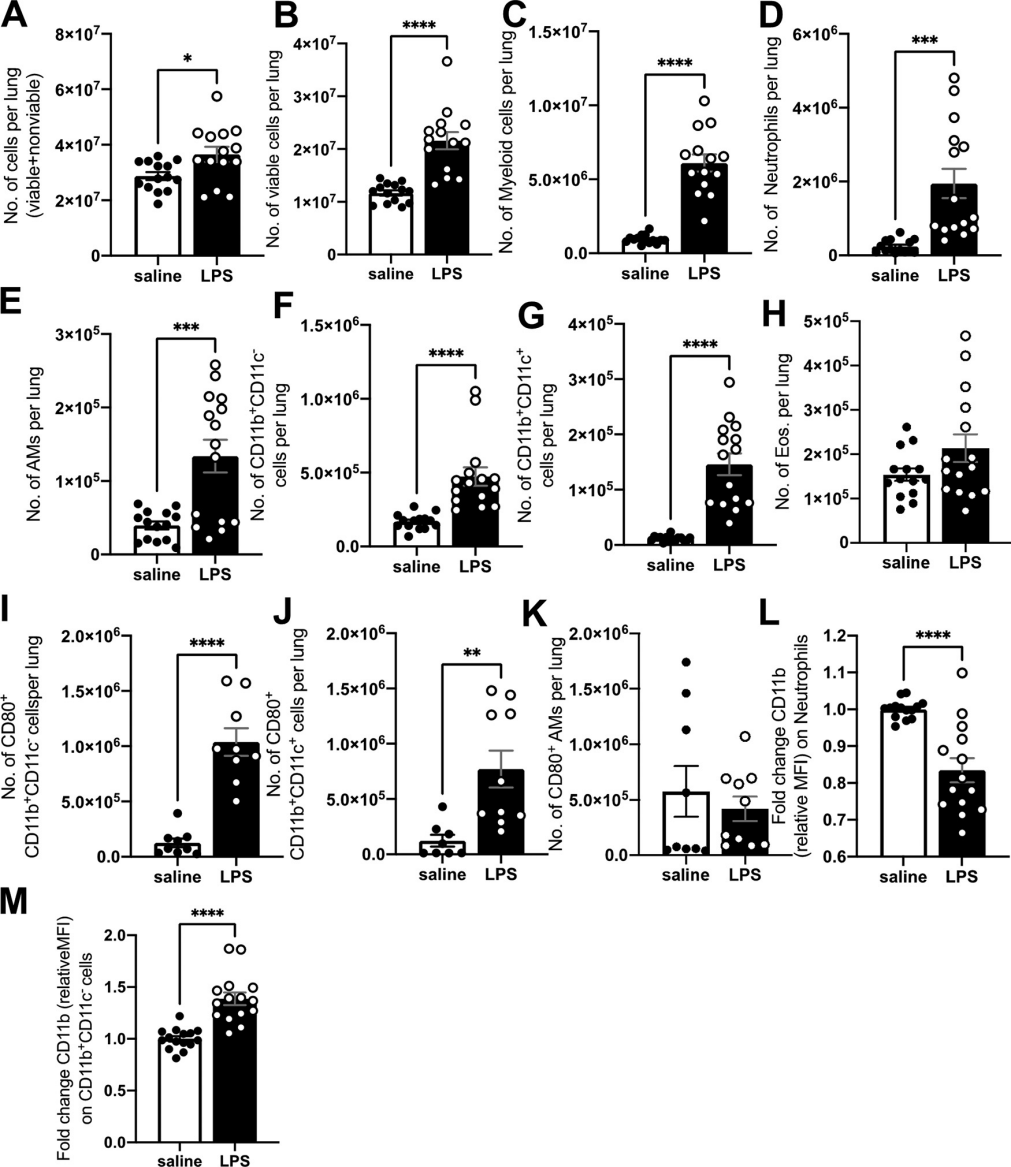
Day 0 lung cell profiles. Single-cell lung suspensions were obtained at day 0 (uninfected) from male BALB/c mice. (A and B) Total number of cells (viable+nonviable) (A) and total number of viable cells (B) per lung. (C to M) Flow cytometric analysis of single-cell suspensions. Gating was done as described in Fig. S3. (C to H) Number (No.) of myeloid cells (CD45^+^ SSC-L^hi^) (C), neutrophils (CD11b^+^Ly6G^+^) (D), alveolar macrophages (AMs) (Ly6G^lo/neg^CD11c^+^SiglecF^+^) (E), CD11b^+^ cells (Ly6G^lo/neg^CD11b^+^ CD11c^−^) (F), CD11b^+^ CD11c^+^ cells (Ly6G^lo/neg^CD11b^+^ CD11c^+^) (G), and eosinophils (eos.) (Ly6G^lo/neg^CD11c^−^SiglecF^+^) (H) per lung. Absolute numbers of cells per lung are shown. (I to K) No. of CD80^+^ CD11b^+^ cells (I), CD80^+^ CD11b^+^ CD11c^+^ cells (J), and CD80^+^ AMs (K) per lung. (L to M) Relative fold change CD11b expression (MFI) on neutrophils (L) and CD11b^+^ cells (M) relative to saline. Data are representative of 2 to 3 independent experiments with 4 to 5 mice in each group. Unpaired Student's *t* test, *, *P* < 0.05; **, *P* < 0.01; ***, *P* < 0.001; ****, *P* < 0.0001.

To determine the cellular mechanism behind the early M. tuberculosis control in LPS mice, we analyzed lung cellular profiles at day 0, prior to infection. LPS mice had more total cells (Fig. 2A), more total viable cells (Fig. 2B), and more myeloid cells (CD45^+^ SSC^hi^) per lung (Fig. 2C). We used side scatter screening to obtain an estimate of myeloid cells in the lung, but contamination of other cell types in this specific population cannot be fully ruled out (27). Flow gating schemes and representative images of LPS- or saline-treated mouse profiles are shown in Fig. S3. Further analysis showed that LPS mice had larger amounts of neutrophils (CD11b^+^ Ly6G^hi^) (Fig. 2D), alveolar macrophages (AMs) (Ly6G^lo/neg^ CD11c^+^ SiglecF^+^) (Fig. 2E), and cells that both singularly expressed CD11b (Ly6G^lo/neg^ CD11b^+^ CD11c^−^) (Fig. 2F) and dually expressed CD11b and CD11c (Ly6G^lo/neg^ CD11b^+^ CD11c^+^) (Fig. 2G) in LPS mice. Eosinophil (eos.) (Ly6G^lo/neg^ CD11c^−^ SiglecF^+^) numbers were relatively unchanged (Fig. 2H). Regarding CD11b^+^ cells, our hierarchical gating scheme attempted to identify cells of monocyte origin and macrophages, but as these cells lacked further characterization, we subsequently refer to these cells as CD11b^+^ cells. CD80 is upregulated on macrophages after activation (28), and we found higher numbers of CD80^+^ CD11b^+^ CD11c^−^ cells and CD80^+^ CD11b^+^ CD11c^+^ cells (Fig. 2I and J) in LPS mice. No differences were seen in CD80^+^ AMs (Fig. 2K). We also determined CD11b expression on neutrophils and CD11b^+^ CD11c^−^ cells and found a significantly lower level of CD11b mean fluorescence intensity (MFI) on neutrophils and a significantly higher CD11b MFI on CD11b^+^ cells (Fig. 2L and M) in LPS mice, suggesting higher CD11b surface expression on the CD11b^+^ cells and less on neutrophils from LPS mice.

### Elevated numbers of myeloid cells in LPS mice persist at 1 week but normalize by 2 weeks after M. tuberculosis infection.

**FIG 3:**
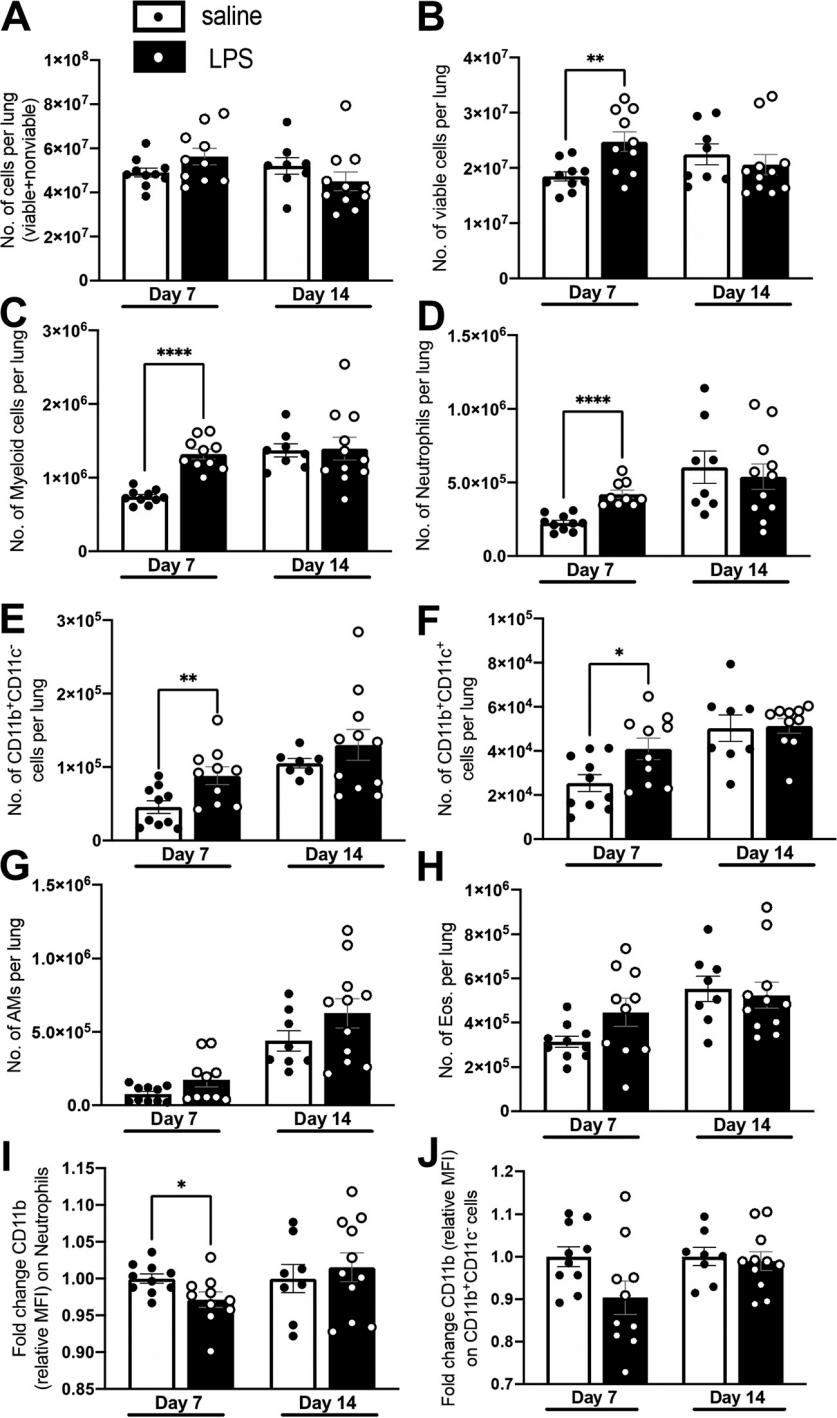
Lung cell profiles at 7 and 14 dpi. Lung single-cell suspensions prepared at the indicated time point postinfection as described from male and female BALB/c mice. (A and B) Total number of cells (viable+nonviable) (A) and total number of viable cells (B) per lung. (C to J) Flow cytometric analysis of single-cell suspensions. Gating was done as described in Fig. S3. (C to H) Number (No.) of myeloid cells (CD45^+^ SSC-L^hi^) (C), neutrophils (CD11b^+^ Ly6G^+^) (D), CD11b^+^ cells (Ly6G^lo/neg^ CD11b^+^ CD11c^−^) (E), CD11b^+^ CD11c^+^ cells (Ly6G^lo/neg^ CD11b^+^ CD11c^+^) (F), AMs (Ly6G^lo/neg^ CD11c^+^ SiglecF^+^) (G), and eos. (Ly6G^lo/neg^ CD11c^−^ SiglecF^+^) (H) per lung. (I to J) Relative fold change of CD11b expression (MFI) on neutrophils (I) and CD11b^+^ cells (J) relative to saline. Data are representative of 2 independent experiments with 4 to 5 mice in each group. Unpaired Student's *t* test, *, *P* < 0.05; **, *P* < 0.01; ****, *P* < 0.0001.

We next determined when, and if, the levels of myeloid cells normalized in M. tuberculosis-infected LPS mice relative to M. tuberculosis-infected saline mice. Total number of cells showed no differences in M. tuberculosis-infected LPS mice at 7 and 14 dpi (Fig. 3A). Total numbers of viable cells, however, were elevated at 7 dpi, but no differences were seen between groups at 14 dpi (Fig. 3B). The same trend was observed in total myeloid cells at 7 and 14 dpi (Fig. 3C). Flow cytometric analysis of specific myeloid cell populations showed higher numbers of neutrophils (Fig. 3D), CD11b^+^ CD11c^−^ cells (Fig. 3E), and CD11b^+^ CD11c^+^ cells (Fig. 3F) in M. tuberculosis-infected LPS mice at 7 dpi, which normalized to M. tuberculosis-infected saline mice by 14 dpi. AMs (Fig. 3G) and eos. (Fig. 3H) showed no significant differences at 7 or 14 dpi. Elevated cell numbers in LPS mice compared to saline mice at 7 dpi, but not 14 dpi, indicated that the inflammatory stimulus from LPS treatment in M. tuberculosis-infected LPS mice was acute. Furthermore, we observed CD11b MFI differences for both neutrophils and CD11b^+^ CD11c^−^ cells at 7 dpi, but only the former was significant (Fig. 3I and J). At 14 dpi, CD11b MFI differences were normalized on neutrophils and CD11b^+^ CD11c^−^ cells from LPS and saline mice (Fig. 3I and J). These results show that the M. tuberculosis-infected LPS mice were under acute inflammation at the time of infection, and the cellular events responsible for the early protection against M. tuberculosis likely occurred within the first week postinfection. This was supported by the lower number of CFU in LPS mice as early as 7 dpi (Fig. 1C).

### CD11b^+^ cells from LPS mice do not contribute to *in vitro* control of M. tuberculosis.

**FIG 4:**
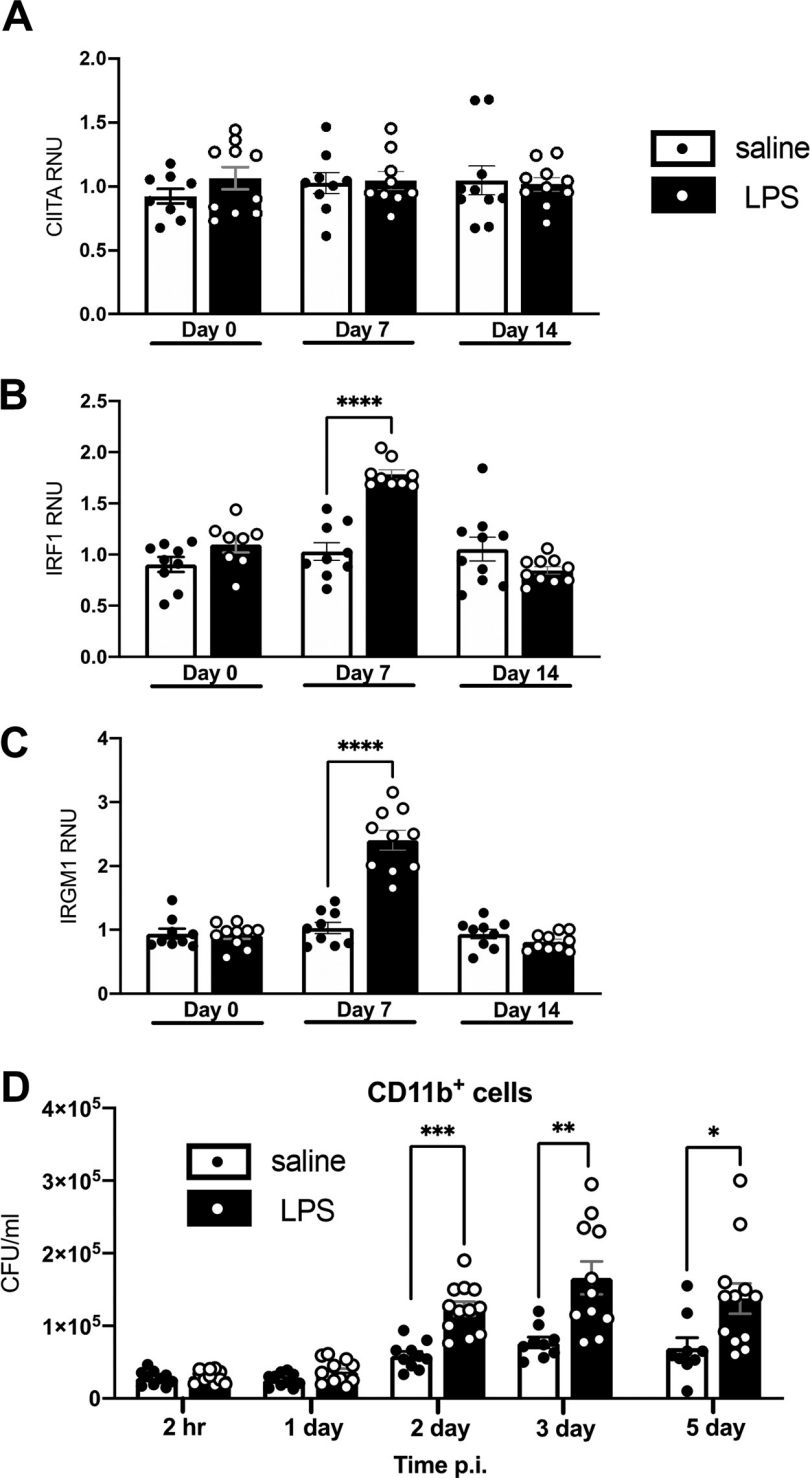
Lungs cells from LPS mice are transiently activated, while CD11b^+^ cells are detrimental to *in vitro* M. tuberculosis infection. (A to C) At the indicated time point, RNA was isolated from pulmonary macrophages from female BALB/c mice and quantified using quantitative real-time PCR. CIITA (A), IRF1 (B), and IRMG1 (C) levels are shown. Data are expressed as relative numerical units (RNU) relative to saline mice and is representative of 2 independent experiments of 4 or 5 mice in each group. (D) CD11b^+^ cells were isolated from uninfected female BALB/c mice and infected *in vitro* with M. tuberculosis. Numbers of CFU at the indicated time point are shown. Data are expressed as CFU/ml and are represented by 3 independent experiments with 2 to 5 samples in each group. Unpaired Student's *t* test, *, *P* < 0.05; **, *P* < 0.01; ***, *P* < 0.001; ****, *P* < 0.0001.

To determine the contribution of macrophages in the early M. tuberculosis control by LPS mice, we measured mRNA expression levels of several inflammatory transcription factors (CIITA, IRF1, and IRGM1) (29–32) from adherent lung cells at day 0 (uninfected) and 7 and 14 dpi. Flow cytometric analysis found the majority of adherent cells to be AMs, with some CD11b^+^ cells (total adherent cells, referred to as pulmonary macrophages) (∼60% AMs, ∼6% CD11b^+^ cell; Table S1). We observed no differences in CIITA mRNA levels at any time point (Fig. 4A), whereas IRF-1 and IRGM-1 showed an increase in M. tuberculosis-infected LPS mice at 7 dpi only (Fig. 4B and C). We concluded that pulmonary macrophages in LPS mice transiently upregulated inflammatory transcription factors after day 0.

We next purified CD11b^+^ cells at day 0 (uninfected) from LPS or saline mice based on CD11b positive magnetic selection (∼94% CD11b^+^ cells; Table S1), infected *in vitro* with green fluorescent protein (GFP)-expressing M. tuberculosis Erdman, and determined CFU numbers at 2 h and 1, 2, 3, and 5 dpi. We observed no differences between groups at 2 h and 1 dpi, but 2, 3, and 5 dpi showed significantly higher M. tuberculosis CFU numbers in CD11b^+^ cells isolated from LPS mice (Fig. 4D). This suggested that the CD11b^+^ cell infiltrates we saw in LPS mouse lungs at day 0 do not contribute to the early control we see after *in vivo* infections and may be detrimental.

### Neutrophils are responsible for enhanced control of M. tuberculosis infection in LPS mice.

**FIG 5:**
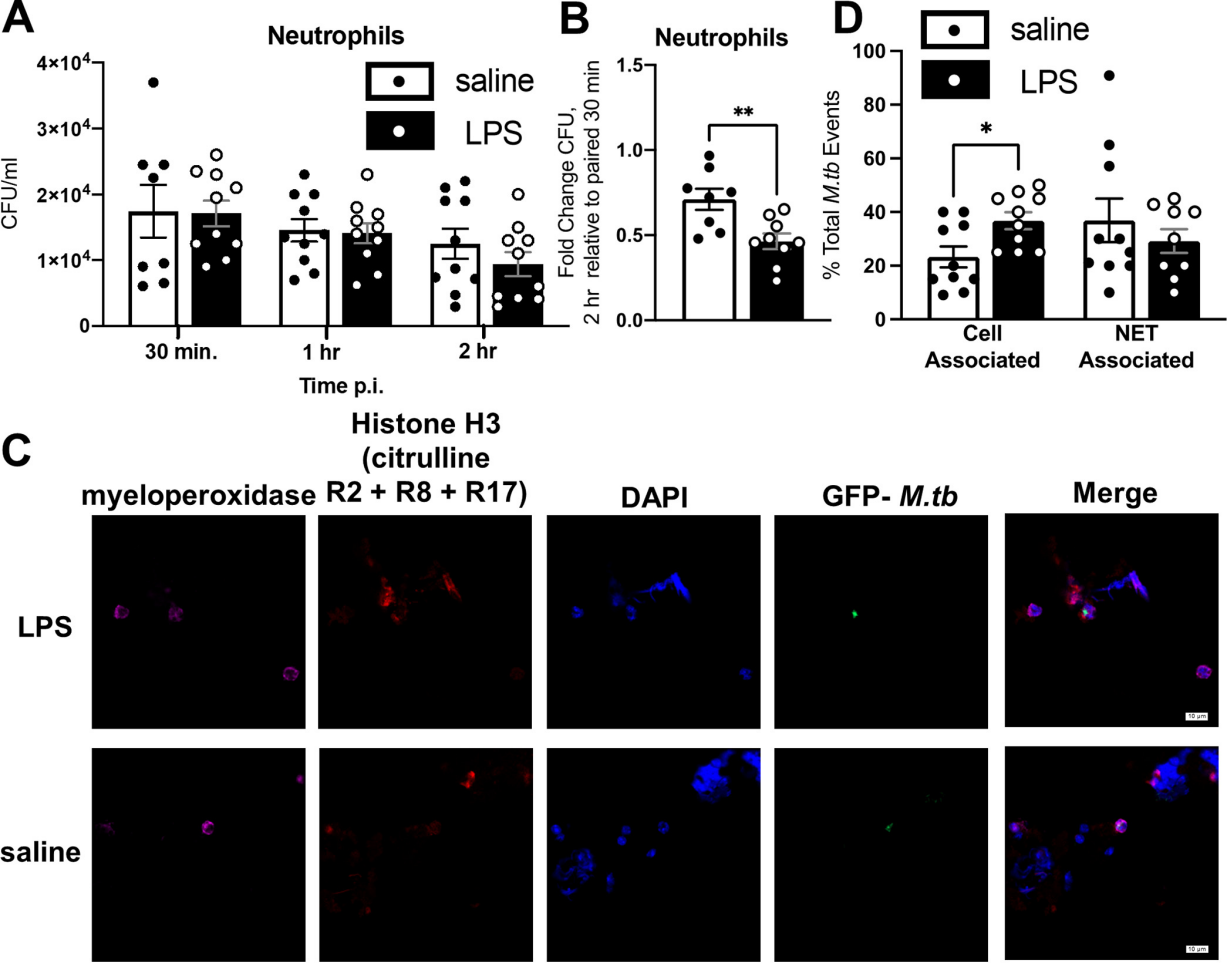
Neutrophils from LPS mice are capable of increased M. tuberculosis killing. Neutrophils were isolated from uninfected female BALB/c mice as described. (A) Number of CFU during *in vitro* infections. (B) Fold change of CFU numbers over 2 h of neutrophil infection, calculated relative to paired 30-min data. (C) Immunocytochemistry of neutrophil infections. Pink, myeloperoxidase; red, citrullinated histone H3 (R2+R8+R17); blue, DAPI; green, GFP M. tuberculosis. Representative images of a GFP-M. tuberculosis event colocalized with an intact cell (neutrophil) are shown. (D) The location of GFP-M. tuberculosis in each well, expressed as percentage of total M. tuberculosis events. Slides were analyzed in a single-blinded manner. Nineteen to 22 GFP-M. tuberculosis events were analyzed per well. Cell-associated, GFP-M. tuberculosis colocalized with an intact cell; NET associated, GFP-M. tuberculosis colocalized with a neutrophil NET. Data are representative of 2 independent experiments with 4 to 5 samples in each group. Unpaired Student's *t* test, *, *P* < 0.05; **, *P* < 0.01.

LPS treatment also increased neutrophils in the lung (Fig. 2D). To determine the potential involvement of neutrophils in control of M. tuberculosis infection in LPS mice, we isolated neutrophils via Ly6G positive magnetic selection at day 0 (uninfected) (∼98% neutrophils; Table S1) and infected the purified cells *in vitro* with GFP-M. tuberculosis Erdman. At 30 min, 1 h, and 2 h postinfection, we determined CFU numbers (Fig. 5A). While at each time point there was no significant difference between CFU numbers in LPS and saline mice, we elected to analyze the differences between samples at the different time points by calculating the fold change of the 2-h CFU time point over the 30-min CFU time point among samples originating from the same mouse. We found a greater fold change of LPS neutrophil CFU numbers (2 h versus 30 min) than saline neutrophil CFU numbers (Fig. 5B), suggesting a greater magnitude of M. tuberculosis control in LPS neutrophils. When we analyzed the infection using single-blinded immunocytochemistry (ICC) microscopy (Fig. 5C), we found no differences in M. tuberculosis association with neutrophil extracellular traps (NETs), but the numbers of M. tuberculosis colocalizing with intact neutrophils was significantly increased in neutrophils from LPS mice compared to saline mice (Fig. 5D).

**FIG 6:**
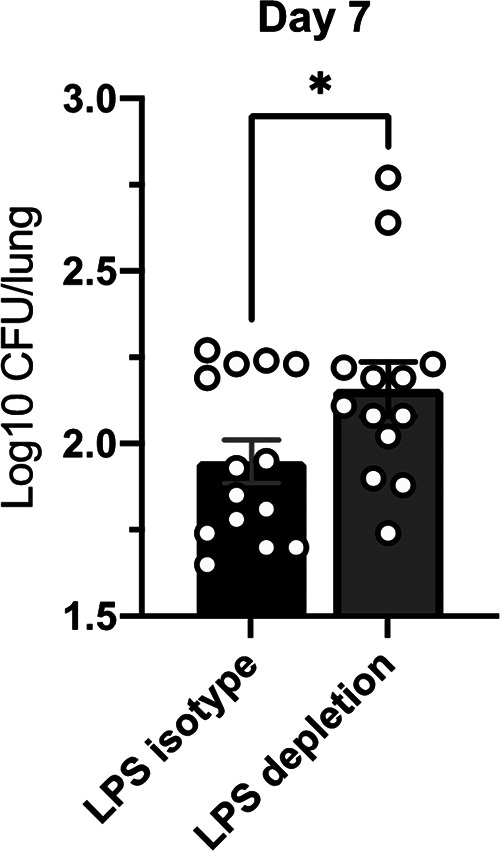
Neutrophils drive control in LPS mice. Neutrophils were depleted from LPS and saline female BALB/c mice using monoclonal antibodies against Ly6G as described in the text. Numbers of CFU at 7 dpi in LPS mice injected with depletion or isotype antibodies are shown. Data are representative of 4 independent experiments with 3 to 4 mice in each group. Unpaired Student's *t* test, *, *P* < 0.05.

To confirm that LPS neutrophils were responsible for conferring increased control of M. tuberculosis, we partially depleted neutrophils over the course of *in vivo* infection (∼50% depletion efficiency; Table 1). At 7 dpi, M. tuberculosis-infected LPS mice depleted of neutrophils showed higher levels of M. tuberculosis CFU (∼0.25 log_10_) than M. tuberculosis-infected LPS mice injected with the isotype control antibody (Fig. 6), although variability was seen, as some mice were observed with no CFU differences between the groups. Increased killing *in vitro* (Fig. 5), together with these results, suggests neutrophils were involved in the increased early control of M. tuberculosis infection in LPS mice *in vivo*, although the contribution of other cell types in this early control phenomenon cannot be ruled out.

**TABLE 1:** Neutrophils present in mice lungs depleted of neutrophils at day 0^*a*^

Sample	No. of neutrophils ± SD (if applicable)	Depletion efficiency versus LPS or saline isotype (%)	Fold change versus saline isotype
LPS isotype	7.08 × 10^6^		32.32
LPS depletion	3.58 × 10^6^ ± 4.25 × 10^5^	50.56	16.34
Saline isotype	2.19 × 10^5^		1.00
Saline depletion	1.11 × 10^5^	50.68	0.50

a The average absolute numbers of neutrophils per lung at day 0 are shown. Saline mice were included as a reference. Data are representative of 1 independent experiment with 1 to 2 samples in each group.

## DISCUSSION

Inflammation can affect the control of TB disease (33–35), yet the impact of basally increased inflammation (occurring in the elderly, persons with lung coinfections, those with environmental insult, etc.) at the time of M. tuberculosis infection has received limited attention (36–38). To investigate this, we developed a mouse model to test if acute inflammation induced by a short-term low-level LPS delivery could alter control of M. tuberculosis infection. LPS was chosen as a commonly used stimulus to induce acute inflammation in the mouse model (39–43), primarily signaling through toll-like receptor 4 (TLR4) to generate an inflammatory response (44). Although LPS signaling may not be representative of other inflammatory states generated by signaling through other TLRs and pathogen recognition receptors, it is a well-characterized acute inflammatory stimulus with predictable cytokine responses and a multitude of cellular activations and influxes (45–47). Mice injected with LPS established an acute pulmonary and systemic inflammatory state and an influx of neutrophils and CD11b^+^ cells into the lungs prior to infection, which can be expected after an acute inflammatory stimulus such as LPS (48). After M. tuberculosis infection, LPS mice had lower M. tuberculosis burden than controls as early as 7 dpi and continuing up to 6 months postinfection. *In vitro* and *in vivo* studies demonstrated that neutrophils were involved in the enhanced control of M. tuberculosis in LPS mice. These findings suggest that acute inflammation can confer protection against M. tuberculosis infection in the mouse model.

Our results suggest that an acute inflammatory state at the time of M. tuberculosis infection can be protective against M. tuberculosis. Examples of this in the literature include studies in old mice, which are chronically inflammatory, both in the periphery and the lung (49–51). This chronic inflammatory status makes old mice display an early control of M. tuberculosis infection compared to their younger counterparts (24, 52, 53). This early control is considered a consequence of the chronic but moderate inflammatory state at the time of infection in old mice, as work from our group has indicated (8–11), and we showed that innate cells in the lungs of old mice are preactivated and behave differently in response to M. tuberculosis (49). In contrast to LPS mice (acute model), old mice (chronic model) cannot sustain control, likely due to their reported reduced adaptive immune function (54, 55), and old mice succumb to the infection earlier than young mice (52, 56). Adaptive immune function was not investigated in LPS mice, but as we observed decreased M. tuberculosis burden in LPS mice up to 6 months p.i., the longest time point we tested, the initial innate response against M. tuberculosis in LPS mice was enough to maintain protection long term in our model. Beyond the initial decrease in bacterial burden, however, we do not know how LPS mice were able to maintain a lower level of M. tuberculosis long term. It may be due to alteration of the cellular profiles during long-term infection or by restructuring of lung granulomas. Alternatively, the neutrophil influx observed in LPS mice may activate other cells, such as dendritic cells, enhancing their actions to keep the M. tuberculosis level lower (57). The simplest explanation may be that LPS and saline mice, although given the same dose of M. tuberculosis, have a different bacterial burden at 14 dpi, the starting point when adaptive immunity against M. tuberculosis can first be detected in the lungs (58, 59), and this may be enough for LPS mice to maintain their bacterial burden using typical adaptive immunity. Resembling this effect, mice infected with different doses of M. tuberculosis show higher or lower levels of M. tuberculosis burden at late stages of the infection, depending on a higher or lower initial infectious dose, respectively (60, 61). It is interesting, though, that LPS mice do not lose their early control, either back to the level of saline mice, or show a worsening of burden. This implies their early inflammatory stimulus causes no negative long-term effects.

In human active TB, neutrophils are the most infected phagocytic cell and can provide a niche for M. tuberculosis persistence and survival (62). Furthermore, neutrophil influx to the lungs is associated with worse TB disease in patients (63). Neutrophils enter the lung in high numbers after M. tuberculosis infection, and are typically reported to kill M. tuberculosis via phagocytosis and subsequent killing as well as extracellular killing mechanisms (degranulation) (64, 65). Neutrophil NETs can also contribute to control via slowing of M. tuberculosis growth and M. tuberculosis death (66) but are also reported to contribute to the worsening of disease (67). The amount of conflicting information on neutrophils in M. tuberculosis infection suggests the role of neutrophils is context dependent. Indeed, rodent studies show that neutrophils play a beneficial role in early infection but a negative role at later stages (37, 38, 68, 69). A study of LPS-induced lung neutrophilia in rats showed reduced M. tuberculosis burden if LPS was delivered prior to infection, which was negated following neutrophil depletion (38). LPS delivery 10 days post-M. tuberculosis infection, however, had no effect. Our results from LPS mice corroborate these findings and suggest that neutrophils can play a beneficial role in early infection, if they are increased in number and primed prior to the arrival of M. tuberculosis to the lungs. In our own neutrophil depletion studies, however, we recognize that there was only a small increase (∼0.25 log_10_) of M. tuberculosis CFU numbers in LPS mice depleted of neutrophils compared to LPS mice with no neutrophil depletion. Additionally, some neutrophil-depleted LPS mice showed no difference in CFU at all. Complete depletion of neutrophils is challenging (66, 70–73) and we achieved 51% depletion in LPS mice, still representing a 16.34-fold increase in neutrophils compared to the basal levels seen in saline mice, which likely influenced the modest changes in CFU numbers. It is accepted that depletion of neutrophils in mice with the 1A8 clone is difficult for a variety of reasons (70). After depletion, the bone marrow generates neutrophils to maintain homeostasis (74), which, coupled with the LPS stimulus, likely caused abundant neutrophils present in the periphery to be depleted, resulting in incomplete depletion. Although we cannot conclude that neutrophils are the singular determinant of early control in LPS mice, we can speculate from their known involvement in inflammation (63, 75–77) that they contribute to the inflammation-mediated early control of M. tuberculosis infection in LPS mice.

The contribution of neutrophils in mediating some control of M. tuberculosis infection in LPS mice was supported by our *in vivo* flow cytometry data, where neutrophil numbers were increased most in lungs of LPS mice compared to other cells. Our *in vitro* work also suggested that neutrophils from LPS mice phagocytose and kill M. tuberculosis more effectively, although we observed differences in CFU numbers only after data pairing, possibly due to the low sensitivity of the CFU method. Infections with a multiplicity of infection (MOI) of 5:1 indicated that not all the bacteria were taken up by cells, possibly contributing to enumeration issues in this *in vitro* model. Additionally, during ICC experiments of neutrophils, we observed differences in M. tuberculosis-neutrophil colocalization between saline and LPS mice at 30 min of infection but no differences in CFU numbers during the 30 min time point itself. We believe this discrepancy results from the time difference between phagocytosis of the bacterium and its eventual killing (78). We suspect seeing early increases in colocalization of M. tuberculosis with LPS neutrophils sets up LPS neutrophils to show lower CFU numbers during the whole infection.

We observed less CD11b expression on LPS neutrophils, a component of complement receptor 3 (CR3) (79). CR3 is one of the major phagocytic receptors for M. tuberculosis and can be activated by M. tuberculosis on neutrophils, although it is not known if neutrophils directly use CR3 to phagocytose M. tuberculosis (79–81). Because we observed no negative impact in the colocalization of M. tuberculosis and LPS neutrophils, we can conclude that the lower levels of CD11b expression on LPS neutrophils had a limited effect. Interestingly, a recent experiment showed that lowering levels of CD11b expression on neutrophils resulted in increased protection against M. tuberculosis in a mouse model, although this was associated with decreased neutrophil accumulation in the lungs (82). Given the complicated and often disparate roles of neutrophils shown in the literature, more studies are needed to address the role of neutrophils in differing states of M. tuberculosis infection, especially in cases where neutrophil behavior is altered (37, 38, 68, 69, 82).

The other cell type we interrogated as potentially being responsible for control in LPS mice was CD11b^+^ cells. Our hierarchal flow cytometry gating scheme attempted to phenotype cells of monocyte origin (CD11b^+^ cells) (83); however, we were unable to specifically differentiate between monocytes, macrophages, and CD11b^+^-expressing dendritic cells. For simplicity, we labeled all cells in this gate CD11b^+^ cells, but they are likely of monocyte origin and make up a mixture of macrophages, monocytes, and dendritic cells (84). When we tested the cells *in vitro*, our results suggested CD11b^+^ cells were not major contributors to the increase in M. tuberculosis control seen LPS mice. As M. tuberculosis infections progress in a mouse, macrophages are known to be essential for infection control, and the transition of M. tuberculosis to interstitial macrophages has been shown to beneficial (85, 86). However, interstitial macrophages can also provide M. tuberculosis a niche for survival (87–89). A recent experiment on an alveolar macrophage subset showing monocytic markers in old mice displayed worse control of M. tuberculosis (90), and in humans and mice infected with M. tuberculosis, a monocyte influx to the lungs correlates with worse TB disease outcome (91, 92). These findings substantiate the results from our *in vitro* infections of CD11b^+^ cells in LPS mice, where worse control of M. tuberculosis growth was seen *in vitro*. The exact consequence of the alterations in CD11b^+^ cells in LPS mice was not determined from the experiments we performed. However, it does signify inflammation can affect CD11b^+^ cell function, and we believe our work can be used as a starting point for future studies aimed at assessing the interplay between inflammatory CD11b^+^ cells/macrophages and M. tuberculosis in inflammatory disease states.

Overall, our results demonstrate that basal inflammation at the time of M. tuberculosis infection can positively impact infection outcome. We can conclude that neutrophils played a role in this early control but could not make a definitive conclusion whether CD11b^+^ cells were also directly involved in the early control of M. tuberculosis seen in our model, although our results do suggest that LPS causes changes in these cell types. In a broader sense, our results indicate that inflammation driven by acute insult at the time of M. tuberculosis infection can lead to improved long-term control of infection. In some states, inflammation may work to enhance protection against TB, but in a human host with other factors impacting the immune response (such as HIV coinfection, diabetes, COPD, etc.), these factors may override any positive benefits of inflammation, ultimately resulting in TB susceptibility. The results presented here with LPS mice may have relevance for other coinfections and disease states that cause a short-term (acute) pulmonary inflammation in the lung at the time of M. tuberculosis infection. In these cases, the initial bacterial burden may be lowered during early M. tuberculosis infection and maintained long term, giving rise to differential TB infection outcomes.

## MATERIALS AND METHODS

### Mice.

Specific-pathogen-free male or female BALB/c and male C57BL/6 mice, 11 to 12 weeks old, were purchased from Charles River Laboratories (Wilmington, MA) or The Jackson Laboratory (Bar Harbor, ME). Male and female BALB/c mice were used as indicated in the figure legends. Mice were housed in animal biosafety level 2 (ABSL2) or ABSL3 facilities, in individually ventilated cages, and given sterilized water and standard chow *ad libitum*. Experimental and control mice were housed in separate cages and were acclimatized for at least 1 week before use in experiments. Mice were euthanized by CO_2_ asphyxiation. All procedures were approved by the Texas Biomedical Research Institute Institutional Laboratory Animal Care and Use Committee (IACUC), protocol number 1608 MU.

### LPS mouse model.

Lipopolysaccharides (LPS) (L3129-100MG; Sigma) were injected intraperitoneally (i.p.) with 20 μg/mouse (BALB/c) or 50 μg/mouse (C57BL/6) in 100 μl normal saline (vehicle). Saline injections served as a control. The optimum LPS dose for each individual mouse strain to result in inflammation without persistent morbidity was determined, and mice were injected every 24 h ± 2 h for 4 total injections. Experiments termed day 0 took place 2 h after the fourth injection. Alternatively, mice were infected with M. tuberculosis 2 to 4 h after the fourth injection, as described below. M. tuberculosis-infected mice were injected daily with LPS or saline for 2 more days for a total of 6 injections and rested until the indicated time point.

### M. tuberculosis stocks.

M. tuberculosis Erdman (ATCC 35801) was obtained from the American Type Culture Collection (Manassas, VA). GFP-expressing M. tuberculosis Erdman was kindly provided by Horwitz and colleagues (93). Stocks were grown and delivered via aerosol as previously described (94). For *in vitro* infections, a frozen M. tuberculosis stock was plated onto 7H11 agar (Difco and BBL) supplemented with oleic acid-albumin-dextrose-catalase (OADC) enrichment and incubated for 11 to 13 days at 37°C. A M. tuberculosis single bacterial suspension was generated and diluted to working concentration as described previously (95).

### M. tuberculosis aerosol infection and CFU number calculation.

Mice were exposed to a low-dose aerosol of M. tuberculosis Erdman using an inhalation exposure system (Glas-col) calibrated to deliver 10 to 30 CFU to the lungs of each mouse (96). Calculation of M. tuberculosis (CFU) burden at the indicated time points was performed by plating serial dilutions of whole or partial (superior, middle, inferior, and postcaudal lobes) lung homogenates onto OADC-supplemented 7H11 agar containing Mycobacteria Selectatab (Mast Group, UK). Plates were incubated at 37°C, and CFU were counted after 14 to 21 days and transformed to a log_10_ scale. CFU counts obtained from partial lung homogenates were normalized to the mass of each partial lung.

### Protein ELISA and Luminex analysis.

Organ homogenates were thawed and the resulting supernatants analyzed for cytokines and proteins by enzyme-linked immunosorbent assay (ELISA) (BioLegend, BD, and R&D) and Luminex (R&D) according to the manufacturers’ instructions. Protein levels were normalized to lung mass.

### Lung cell isolation.

As described previously, mice were euthanized and lungs perfused with 10 ml 1× Dulbecco's phosphate-buffered saline without calcium or magnesium (Gibco) (PBS) containing 50 U/ml heparin (Sigma) and placed into 2 ml complete Dulbecco’s modified Eagle’s medium (c-DMEM), DMEM (10-017-CV, Corning), 500 ml supplemented with filter-sterilized 5 ml HEPES buffer (1 M; Sigma), 10 ml MEM nonessential amino acid solution (100×; Sigma), 5 ml penicillin-streptomycin (pen/strep) (100×; Sigma), 660 μl 2-mercaptoethanol (50 mM; Sigma), and 45 ml heat-inactivated fetal bovine serum (FBS) (Atlas Biologicals). A single-cell suspension was obtained using enzymatic digestion (49). Residual erythrocytes were lysed using Gey’s solution (8 mM NH_4_Cl, 5 mM KHCO_3_ in water), passed through a 40-μm strainer, and suspended in c-DMEM. The total number of viable cells was determined with acridine orange and propidium iodide (AO/PI) staining and counted on a Cellometer K2 cell counter (Nexcelom Bioscience).

### Flow cytometry.

Lung single-cell suspensions were washed once with 1× PBS and stained with Zombie Aqua fixable viability kit (1:100; BioLegend) for 15 min at room temperature (RT) in the dark. After incubation, cells were washed once with 1× PBS and incubated in TruStain FcX-Fc block (BioLegend) for 15 min at 4°C in the dark. Fc block was diluted in deficient RPMI (dRPMI; RPMI 1640 supplemented with HEPES and 1 g/liter sodium azide [ThermoFisher Scientific]) with 10% heat-inactivated FBS (dRPMI+FBS). Fc block was then removed and cells incubated in antibody cocktails (CD45.2, peridinin chlorophyll protein [PerCP]-cyanine5.5, clone 104; Ly6G, Alexa Fluor 488, clone 1A8; CD11b, allophycocyanin [APC]-cyanine7, clone M1/70; CD11c, APC, clone N418; Siglec-F, Brilliant Violet 421, clone S17007L; CD80, phycoerythrin [PE], clone 16-10A1; BioLegend) diluted in dRPMI+FBS for 20 min at 4°C in the dark. After staining, cells were washed once in dRMPI+FBS. For experiments completed at day 0 (uninfected mice), cells were fixed in 2% paraformaldehyde (PFA) for 15 min at RT in the dark and washed once more in dRPMI+FBS. For experiments completed after infection, cells were fixed in 4% PFA for 30 min at RT in the dark for ABSL3 removal and washed twice in dRPMI+FBS. All stained and fixed cells were suspended in dRPMI+FBS and stored at 4°C in the dark until data acquisition on a Beckman Coulter CyAn flow cytometer. Data analysis was performed using FlowJo v10 (BD Biosciences). Total numbers of specific cell populations per lung were calculated using the percentage of each population in the parent gate for absolute quantification multiplied by the total number of viable cells isolated from each sample.

### Collection of adherent lung cells.

Lung single-cell suspensions were incubated in tissue culture plates for 1 h at 37°C, 5% CO_2_ (49). Plates were washed with c-DMEM to remove nonadherent cells. For RNA isolation, TRIzol (ThermoFisher Scientific) was added to the plates and vigorously pipetted. The TRIzol solution (containing adherent cell RNA) was frozen at −80°C until RNA extraction. For flow cytometric analysis, adherent cells were incubated in trypsin-EDTA (Sigma) for 15 min at 37°C, 5% CO_2_, and c-DMEM was added to stop the reaction. Adherent cells were pooled and suspended in c-DMEM, and viable cell numbers were determined with AO/PI staining as described above. Flow cytometric analysis was performed as described above.

### Real-time PCR.

Frozen TRIzol samples were thawed, and RNA was extracted with chloroform, precipitated using isopropanol and 75% ethanol, and reconstituted in DNase/RNase-free water as previously described (49). cDNA was synthesized with random hexamers using an Omniscript RT kit (Qiagen). cDNA was quantified using TaqMan gene expression probes (ThermoFisher Scientific) and data collected using an Applied Biosystems 7500 real-time PCR instrument. The ΔΔ*C_T_* method was used to quantify relative numerical units (RNU), normalized to endogenous 18S RNA, relative to saline.

### Isolation of purified lung CD11b^+^ cells and neutrophil populations.

Lung single-cell suspensions were prepared from noninfected mice as described above, with the exception that residual erythrocytes were not lysed with Gey’s solution. For isolation of CD11b^+^ cells, lung suspensions from 2 saline-injected mice were pooled, whereas LPS lung suspensions were individually processed. As CD11b is also highly expressed on neutrophils and natural killer (NK) cells (97, 98), we developed a method that enriched lung single-cell suspensions for CD11b^+^ cells by depleting the suspensions of Ly6G^+^ cells (neutrophils) and CD49b^+^ cells (NK cells) via positive selection, followed by positive CD11b magnetic isolation to obtain the leftover cells. Briefly, suspensions were washed once with selection buffer (1× PBS containing 2% FBS, 1 mM EDTA, and 1× pen/strep), suspended at a concentration of 1 × 10^8^ viable cells/ml in 5 ml polypropylene tubes, and first depleted of neutrophils and NK cells using positive magnetic selection, with all incubations carried out in selection buffer using the MojoSort mouse Ly6G selection kit (BioLegend), the EasySep biotin positive selection kit II (Stemcell Technologies), and biotinylated anti-mouse CD49b antibody (25 μg/1 × 10^8^ viable cells; clone DX5; BioLegend) per each kits’ instructions. Following Fc block incubation (Stem Cell), cells were incubated in Ly6G selection antibody and CD49b antibody together, followed by the Stem Cell selection cocktail. Finally, cells were incubated with Ly6G and Stem Cell selection magnetic beads together and selected out using magnets. From the resulting cell suspensions (depleted of neutrophils and NK cells), CD11b^+^ cells were isolated using positive magnetic selection using the EasySep mouse CD11b positive selection kit II (Stem Cell Technologies) according to the manufacturer’s instructions. CD11b^+^ cell viability postisolation was 91.5% ± 1.5% (*n* = 10).

For isolation of lung neutrophils, single-cell suspensions were washed in selection medium and suspended at a concentration of 1 × 10^8^ viable cells/ml. Cells were incubated with normal rat serum (50 μl/1 × 10^8^ viable cells; Stem Cell Technologies and Jackson ImmunoResearch Laboratories) for 5 min at RT. Neutrophils were isolated using the MojoSort mouse Ly6G selection kit (BioLegend) according to the manufacturer’s instructions. Neutrophil viability postisolation was 91.1% ± 4.2% (*n* = 10).

Purified CD11b^+^ cell or neutrophil suspensions were washed and suspended in 100 to 200 μl c-DMEM, and total viable cell numbers were determined with AO/PI staining. Cells were washed with 10 ml antibiotic-free c-DMEM (ABFc-DMEM; c-DMEM without pen/strep added) and suspended at a final concentration of 50,000 viable cells/150 μl in ABFc-DMEM.

### *In vitro* M. tuberculosis infection of CD11b^+^ cells and neutrophils and CFU number determination.

Ninety-six-well plates and 8-well chamber slides were coated with poly-d-lysine (0.1 mg/ml; Gibco) overnight at RT or 2 h at 37°C, washed three times with 1× PBS, and dried before use. Purified cells were plated at 50,000 viable cells per well in 150 μl ABFc-DMEM in a precoated 96-well plate or 8-well chamber slide. Cells adhered to the plate or slide by centrifuging at 300 × *g*, 5 min, 4°C. A 50-μl single-cell suspension of GFP-M. tuberculosis Erdman in ABFc-DMEM, calibrated to deliver M. tuberculosis at a multiplicity of infection (MOI) of 5:1, was then added to the wells.

For CD11b^+^ cells, samples were infected in duplicate. The infection proceeded for 2 h at 37°C, 5% CO_2_ (30 min of shaking followed by 1.5 h of static incubation). After the 2-h infection, cells were washed three times with ABFc-DMEM and incubated under static conditions in ABFc-DMEM until the indicated time point. For CFU enumeration, cells were centrifuged for 300 × *g*, 5 min, 4°C, and washed three times with ABFc-DMEM; liquid was removed after the final wash. Cold distilled water (50 μl) containing 500 μg/ml DNase I (Sigma) was added and incubated for 10 min at RT with periodic agitation. 100 μl of OADC-supplemented 7H9 (Difco) medium and 60 μl of 0.25% sodium dodecyl sulfate (Fisher) in 1× PBS were added and cells incubated for 10 min at RT with periodic agitation. A volume of 75 μl 20% bovine serum albumin (BSA) (Alfa Aesar) in 1× PBS was added, and wells were mixed by pipetting vigorously several times. Resulting solutions were serially diluted, plated, and incubated as described above. Average CFU numbers are expressed as number of CFU/ml.

For neutrophils, samples were infected in triplicate. The infection proceeded for 30 min at 37°C, 5% CO_2_ with constant shaking. After the 30-min infection, cells were washed three times with ABFc-DMEM and incubated under static conditions in ABFc-DMEM until the indicated time point. For CFU enumeration, cells were centrifuged at 300 × *g*, 5 min, 4°C and washed three times with ABFc-DMEM; liquid was removed after the final wash, and 100 μl 0.1% Triton X-100 (Fisher) in 1× PBS was added and cells incubated at RT for 15 min with periodic agitation; 100 μl OADC-supplemented 7H9 medium was added, and wells were mixed by pipetting vigorously several times. Resulting solutions were serially diluted, plated, and incubated as described above. Average CFU numbers are expressed as CFU/ml and as the fold change of number of CFU at 2 h versus 30 min (calculated as 2 h divided by 30 min) among samples that originated from the same mouse. For microscopy studies, cells in 8-well chamber slides were washed three times in ABFc-DMEM following the 30-min infection and then fixed in 4% PFA for 15 min prior to ABSL3 removal and further processing.

### ICC of *in vitro* infections.

Fixed cells on chamber slides were washed three times with 1× PBS and stored at 4°C in the dark until staining. Cells were permeabilized by incubating in 0.5% Triton X-100 for 1 min at RT and washed three times with 1× PBS, 1 min per wash. Blocking buffer (1× PBS with 10% normal donkey serum [Jackson ImmunoResearch Laboratories], 10 mg/ml BSA, and 0.1% Triton X-100) was added and cells incubated for 30 min at 37°C in a humid chamber. Primary antibodies goat anti-human/mouse myeloperoxidase (MPO) (1:100; AF3667; R&D) and rabbit anti-mouse histone H3 (citrulline R2 + R8 + R17) (1:200; ab5103; Abcam), diluted in blocking buffer, were added and incubated for 1 h at 37°C in a humid chamber. Chamber slides were washed three times with 1× PBS, 1 min per wash. Secondary antibodies donkey anti-goat IgG Alexa Fluor 647 (1:10,000; A21447; ThermoFisher Scientific) and donkey anti-rabbit IgG Alexa Fluor 568 (1:1,000; A10042; ThermoFisher Scientific), diluted in blocking buffer, were added and cells incubated for 1 h at 37°C in a humid chamber. Cells were washed as before with three 1-min washes. Chamber slides were then incubated in 4′,6-diamidino-2-phenylindole dihydrochloride (DAPI) (1:5,000; ThermoFisher Scientific) in 1× PBS for 5 min and washed three times in 1× PBS for 5 min with constant shaking. Chamber sides were mounted with coverslips using Prolong diamond antifade mountant (ThermoFisher Scientific) and dried for at least 24 h prior to imaging. Chamber slides were analyzed using a Zeiss LSM 800 confocal microscope. Nineteen to 22 GFP-M. tuberculosis (488 nm) events were counted per well. Histone H3 (citrulline R2 + R8 + R17) and DAPI-smear colocalizations were used to mark neutrophil extracellular traps (NETs), and MPO and circular DAPI were used to mark intact neutrophils. In a single-blinded manner, the locations of GFP-M. tuberculosis events were visually assayed as M. tuberculosis colocalized with an intact neutrophil or M. tuberculosis colocalized with a neutrophil NET. Free M. tuberculosis (outside cells or NETS) was also determined, and no differences were observed between groups. Data are presented as percentage of total GFP-M. tuberculosis events.

### Neutrophil depletion.

Anti-mouse Ly6G (300 μg; clone IA8; BP0075-1; BioXCell) or its isotype control rat IgG2a (clone 2A3; BP0089; BioXCell) in 1× PBS was administered via the i.p. route to LPS/saline mice. Antibody injections began the same day as LPS/saline injections and continued every 48 h. CFU numbers were assessed at 7 days of infection. Single-cell suspensions were isolated at day 0 (uninfected) and analyzed by flow cytometry to assess depletion efficiency. Intracellular Ly6G was used in place of surface Ly6G to account for potential surface antigen masking by the depletion antibody (70). After 2% PFA fixation, intracellular Ly6G was stained in the intracellular staining permeabilization wash buffer (BioLegend) using the manufacturer’s protocol. Ly6G antibodies (Ly6G, PE; clone 1A8; BD Pharmingen) used for intracellular staining were diluted half of what was typically used for surface staining.

### Statistical analysis.

Data analyses, graphing, and statistical analyses were performed using GraphPad Prism 8 and 9 software. Unpaired, two-tailed Student's *t* test was used for two-group comparisons. Statistical significance is reported with asterisks: *, *P* < 0.05; **, *P* < 0.01; ***, *P* < 0.001; ****, *P* < 0.0001. The Grubbs’ test was used to identify outlying data points. Data are presented as individual data points and means ± standard errors of the means (SEM).
