# Supplementary material for: Acute Inflammation Confers Enhanced Protection against Mycobacterium tuberculosis Infection in Mice
Source: Microbiol Spectr. 2021 Jul 7;9(1):10.1128/spectrum.00016-21. doi: 10.1128/spectrum.00016-21 (PMC8552513; doi:10.1128/spectrum.00016-21)
Supplement: SUPPLEMENTAL FILE 1 — Download SPECTRUM00016-21_Supp_1_seq5.pdf, PDF file, 0.7 MB [file spectrum00016-21_supp_1_seq5.pdf]

Supplementary Table 1: Purities of Isolated Cell Populations

Adherent Cell Populations (79.9 ± 0.14% $CD45^+$ cells)					
Sample	AMs	$CD11b^+$ cells	Neutrophils	Eosinophils	Lymphocytes ($CD45^+SSC\text{-}L^{lo}$)
**LPS**	56.40%	9.63%	1.66%	2.47%	29.84%
**saline**	69.40%	3.23%	0.24%	0.50%	26.63%

$CD11b^+$ cells for *in vitro* infections (> 99% $CD45^+$ cells)				
Sample	$CD11b^+$ cells	AMs	Neutrophils	Eosinophils
**LPS**	95.9%	0.26%	2.88%	0.72%
**saline**	93.5%	1.82%	0.5%	1.14%

Neutrophils for *in vitro* infections (> 99% $CD45^+$ cells)	
Sample	Neutrophils
**LPS**	98.7%
**saline**	97.7%

Percentages of total $CD45^+$ cells shown, assessed via flow cytometry. Data are each representative represent of 1 independent experiment with pools of 5 (adherent cells and $CD11b^+$ cells) or 2 (neutrophils) mice in each group.

Supplementary Figure 1. Cytokines in LPS mice at day 0

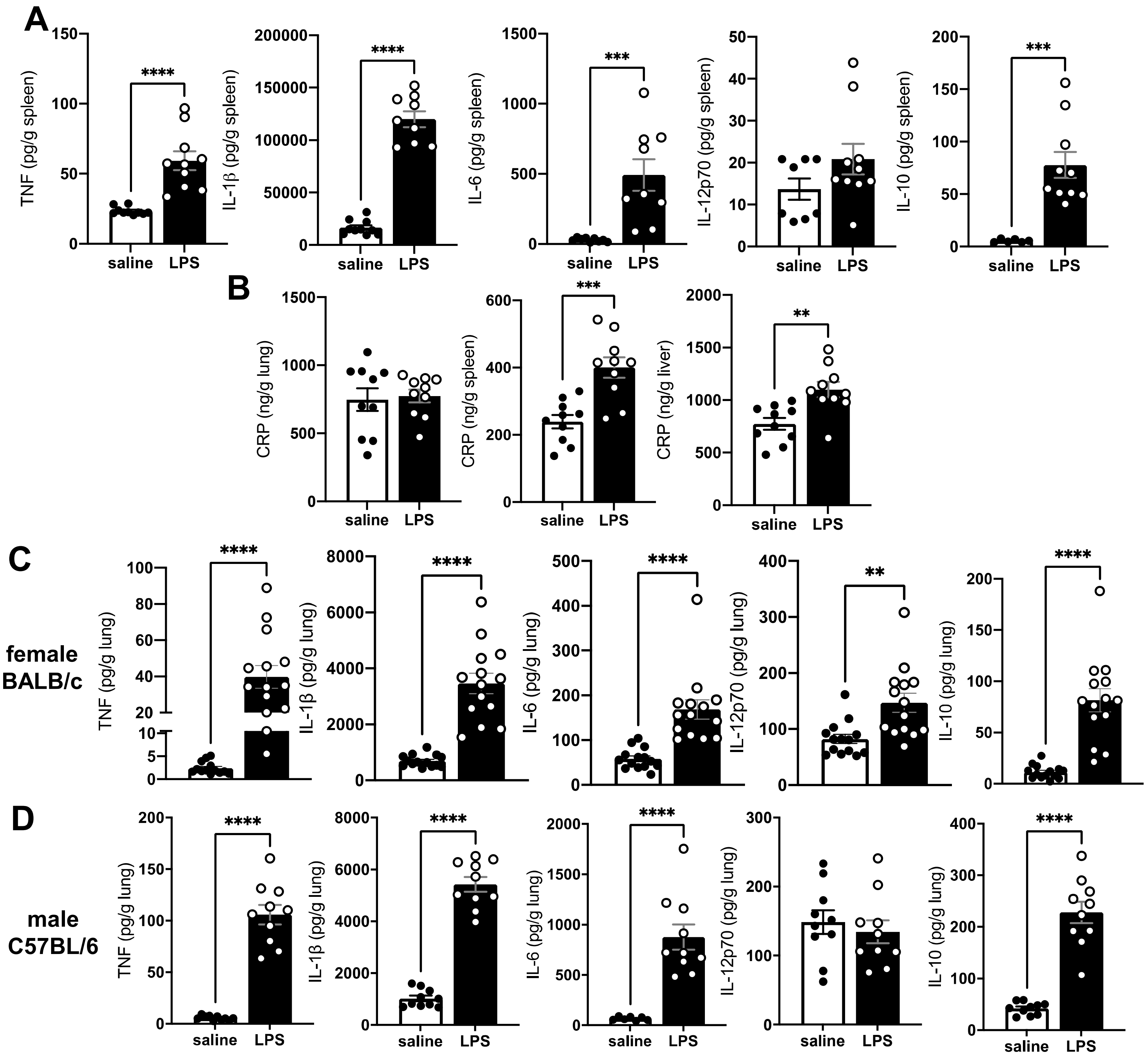

Supplementary Figure 1. Cytokines in LPS mice at day 0. A-B. On day 0, male BALB/c LPS and saline organs were isolated, and protein content determined via Luminex (A) and ELISA (B). TNF, IL-1β, IL-6, IL-12p70, and IL-10 in spleen (A), and CRP in lung, spleen, and liver (B) and are shown. Data is normalized to organ mass **C-D.** On day 0, female BALB/c (C) and male C57BL/6 (D) LPS and saline lungs were isolated, and protein content determined via Luminex. TNF, IL-1β, IL-6, IL-12p70, and IL-10 are shown. Data is normalized to organ mass. Data are representative of 2 independent experiments of 4-5 mice in each group. Unpaired Student's *t* test, $^{**}P<0.01$, $^{***}P<0.001$, $^{****}P<0.0001$.

Supplementary Figure 2. CFUs and Cytokines in infected LPS mice

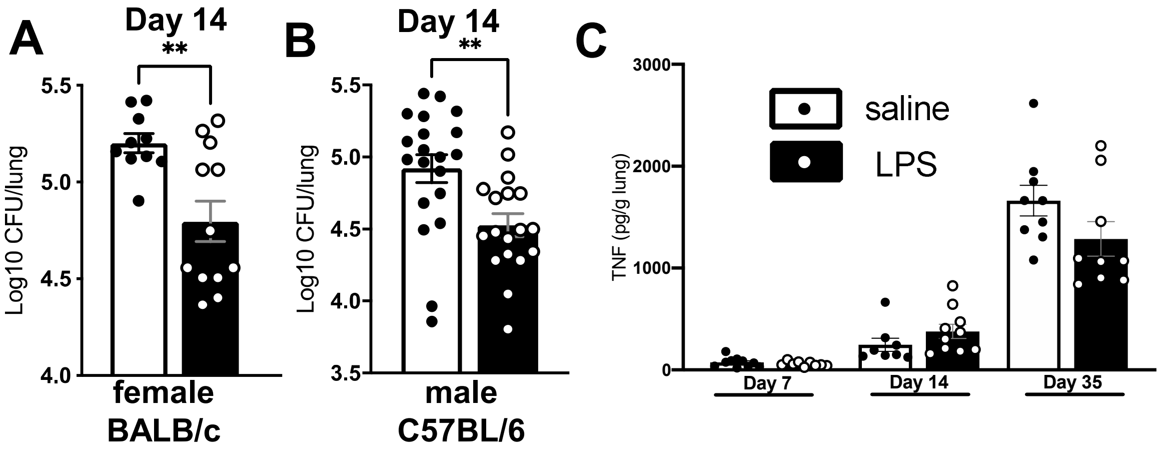

Supplementary Figure 2. Cytokines and CFUs in LPS mice A-B. LPS or saline female BALB/c and male C57BL/6 mice were aerosol-infected *M.tb* Erdman as described. CFU content shown at 14 d.p.i. **C.** LPS or saline male BALB/c mice were aerosol-infected with *M.tb* as described. At the indicated timepoint, protein levels via ELISA of TNF are shown. Data are representative of 2 (A,C) or 4 (B) independent experiments of 2-5 mice in each group. Unpaired Student's *t* test, **P<0.01.

Supplementary Figure 3. Flow Cytometry Gating strategy used

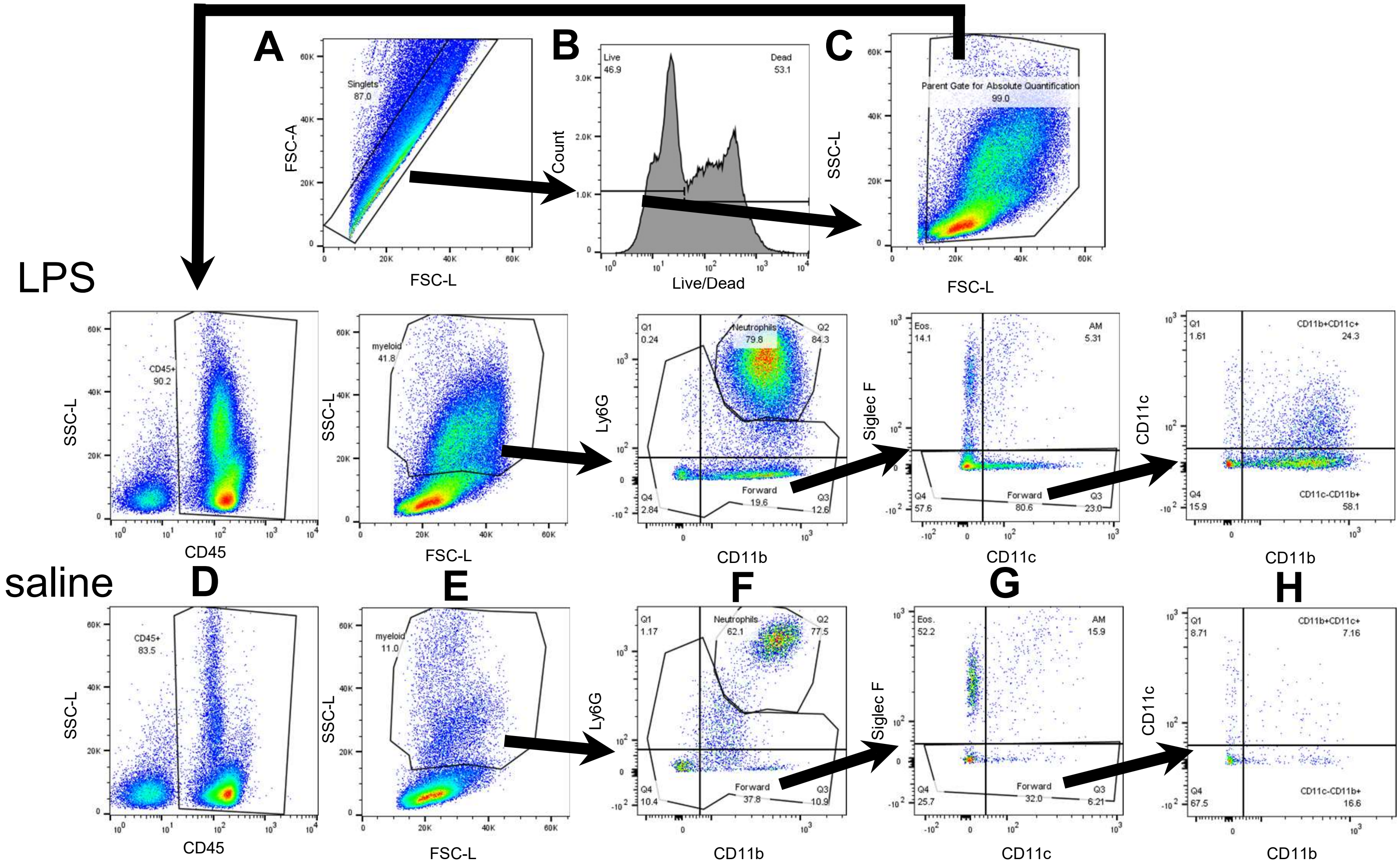

Supplementary Figure 3. Flow Cytometry Gating strategy used. Gating based on fluorescence minus one (FMO) controls. **A-H**. Doublets (A) and dead cells (B) gated out. Parent gate used for absolute number quantification (C) . **D-H** Representative flow cytometry images from LPS and saline mice. CD45$^+$ cells (D) , myeloid cells (CD45$^+$ SSC-L^{hi}) (E)**,** neutrophils (CD45$^+$SSC-L^{hi}CD11b$^+$Ly6G^{hi}) (F), eosinophils (CD45$^+$SSC-L^{hi}Ly6G$^{lo/neg}$SiglecF$^+$CD11c$^-$) and alveolar macrophages (CD45$^+$SSC-L^{hi}Ly6G$^{lo/neg}$SiglecF$^+$CD11c$^+$) (G), and CD11b$^+$CD11c$^+$ cells (CD45$^+$SSC-L^{hi}Ly6G$^{lo/neg}$SiglecF$^-$ CD11b$^+$CD11c$^+$) and CD11b$^+$CD11c$^-$ cells (CD45$^+$SSC-L^{hi}Ly6G$^{lo/neg}$SiglecF$^-$ CD11b$^+$CD11c$^-$) (H).

Supplementary Figure 4. Intracellular flow cytometric analysis of lung neutrophils in mice injected with neutrophil depleting antibody or isotype as described

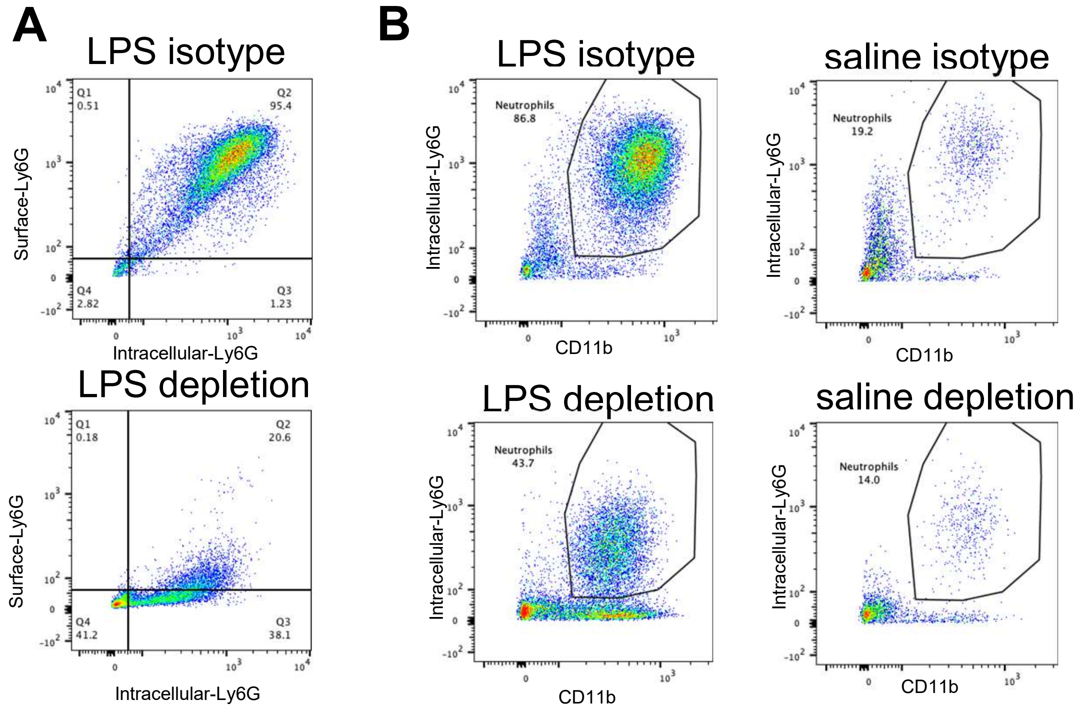

Supplementary Figure 4. Intracellular flow cytometric analysis of lung neutrophils in mice injected with neutrophil depleting antibody or isotype as described. Gating strategy used as in supp. Fig. S3. **A.** Representative images of surface Ly6G vs. intracellular Ly6G in LPS mice. ($CD45^{+}SSC\text{-}L^{hi}CD11b^{+}$), gated from total $CD11b^{+}$ myeloid cells. **B.** Representative images of LPS/saline mice injected with depletion antibody/isotype at day 0. Ly6G is stained intracellularly. ($CD45^{+}SSC\text{-}L^{hi}CD11b^{+}Ly6G^{hi\text{-}intracellular}$), gated from total myeloid cells.
